# Real-World Use of Granulocyte-Colony Stimulating Factor in Patients with Breast Cancer from Alberta, Canada

**DOI:** 10.3390/cancers14246197

**Published:** 2022-12-15

**Authors:** Philip Q. Ding, Brandt J. Newcomer, Winson Y. Cheung

**Affiliations:** 1 Oncology Outcomes, Calgary, AB T2N 4Z6, Canada; 2 Faculty of Medicine and Dentistry, University of Alberta, Edmonton, AB T6G 2R7, Canada; 3 Division of Medical Affairs, Apobiologix, Toronto, ON M9L 2Z7, Canada; 4 Department of Oncology, Cumming School of Medicine, University of Calgary, Calgary, AB T2N 4N2, Canada

**Keywords:** breast cancer, G-CSF, chemotherapy, biosimilar, trends, Canada

## Abstract

**Simple Summary:**

Most chemotherapy regimens used in the setting of non-metastatic breast cancer are myelosuppressive and are associated with toxicities with significant clinical implications, including febrile neutropenia. The use of granulocyte colony-stimulating factor (G-CSF) reduces the severity and duration of febrile neutropenia, following the initiation of myelosuppressive chemotherapy. The practice of G-CSF prophylaxis is a proven form of supportive care that is shifting with the introduction of biosimilars. As published data are limited, we characterized the patterns and predictors of G-CSF use in a large real-world Canadian cohort over an 11-year period. Our results demonstrate that G-CSF use can be further optimized to align with current guidelines and to improve supportive care for patients with breast cancer.

**Abstract:**

Background: There are limited published data in the Canadian healthcare system on the use of granulocyte colony-stimulating factor (G-CSF) among patients with breast cancer. This study characterized real-world G-CSF use during the period surrounding the introduction of filgrastim biosimilar. Methods: Electronic medical records were reviewed retrospectively for patients with breast cancer who received moderately or highly myelosuppressive (neo)adjuvant chemotherapy from 2008 to 2019 in Alberta, Canada. Trends in G-CSF usage were plotted to elucidate temporal variations and multivariable regression models were constructed to identify clinical factors associated with G-CSF use. Results: We included 6662 patients in our analyses. G-CSF was used in 57.1% of patients during their treatment trajectory. Among the 3801 patients who were treated with G-CSF, the majority received pegfilgrastim only (91.5%; *n* = 3477) versus filgrastim only (5.7%; *n* = 217). G-CSF use increased linearly more than two-fold over the 11-year study period. Predictors of G-CSF use included younger age, south zone of residence, higher neighborhood education, inferior disease stage, highly neutropenic risk chemotherapy, and more recent chemotherapy initiation. Conclusions: Despite increasing G-CSF usage over time, an appreciable proportion of patients for whom G-CSF prophylaxis is recommended did not receive it. G-CSF use could be further optimized to align with supportive care clinical guidelines and reduce the impact of neutropenia and its associated complications.

## 1. Introduction

The primary curative intent treatment modality for non-metastatic breast cancer is surgery, but patients with high-risk clinical or pathological features are commonly also offered neoadjuvant or adjuvant therapy as a standard of care. Most chemotherapy regimens used in this setting are myelosuppressive and are associated with a variety of hematological toxicities, including neutropenia and febrile neutropenia (FN). In particular, FN represents the most important dose-limiting toxicity of myelosuppressive chemotherapy, posing significant issues in the management of patients and contributing to a heightened risk of significant morbidity and mortality, increased healthcare resource use, and potential dose modifications with a subsequent failure to achieve optimal relative dose intensity (RDI) [1,2]. Approximately 20–30% of patients with FN require hospitalization, where the mortality rate can be as high as 10% [2]. Moreover, the impact on RDI is a significant concern in the setting of breast cancer management, since failure to achieve an RDI of over 80% may worsen survival outcomes [3,4].

The use of granulocyte colony-stimulating factor (G-CSF) reduces the severity and duration of neutropenia following myelosuppressive chemotherapy, and prophylaxis has been shown to significantly decrease the risk of FN in patients [2,5]. G-CSF may be administered as primary prophylaxis to help prevent a patient’s first episode of FN or as secondary prophylaxis after an episode of FN, where G-CSF is used in subsequent cycles following a previous neutropenic event [6]. G-CSFs, in the form of daily filgrastim or the once-per-cycle, long-acting pegfilgrastim, may be used inconsistently in practice, despite the considerable evidence that they reduce the incidence of FN and related complications [7]. Based on real-world comparative effectiveness studies, pegfilgrastim has been associated with a lower risk of neutropenia-related and all-cause hospitalizations, compared to filgrastim, which may be a result of the under-dosing of short-acting G-CSFs in general practice [8,9,10]; however, current clinical guidelines on myeloid growth factors assume that filgrastim and pegfilgrastim are clinically equivalent.

International guidelines have a high degree of consensus and uniformly recommend prophylactic use of G-CSF when there is a high risk (>20%) of FN [11,12]. FN risk is determined through consideration of both chemotherapy regimen- and patient-specific risk factors. When the chemotherapy risk alone is >20%, G-CSF prophylaxis is recommended. If the chemotherapy risk is moderate (10% to 20%), the presence of one or more patient risk factors may prompt prophylactic G-CSF use. Numerous patient- and disease-related factors are associated with increased overall FN risk, including age ≥ 65 years, advanced disease, poor performance status, presence and number of comorbidities, female sex, prior FN, and laboratory abnormalities (e.g., albumin < 35 g/L, hemoglobin < 12 g/L) [13].

Since the use of supportive care medications in real-world clinical practice varies from the guideline recommendations, and these medications are not uniformly reimbursed in Canada, there are concerns that G-CSF use may be suboptimal or overall under-utilized [14]. In 2017, the filgrastim biosimilar Grastofil was introduced in Alberta, Canada. The reduced costs of biosimilars may diminish some of the barriers associated with G-CSF access. To date, the published data on the use of G-CSF in Canadian patients with breast cancer are lacking, as comprehensive databases linking drug use to patient data and clinical outcomes are not generally available or different to evaluate in a systematic fashion. Additionally, few studies have evaluated the overall use of biosimilar drugs in the years immediately following initial availability, noting that their adoption at the provincial level has varied widely, from a low of 0.1% to a high of 81.6% [15]. The primary objective of this study was to characterize the patterns and predictors of G-CSF use in the period prior to and the initial 2-year period following the introduction of filgrastim biosimilar in Alberta, Canada.

## 2. Methods

### 2.1. Study Design

This was a retrospective, population-based study conducted in Alberta, Canada, which represents the country’s fourth-largest province, with a population of over four million residents. The Alberta Cancer Registry (ACR) was the primary data source for patient demographics, tumor characteristics, and treatment patterns, which were collected prospectively for all patients diagnosed with cancer in the province. Additional data sources included ambulatory care records, physician billing claims, and hospital discharge abstracts, based on previously validated coding algorithms of the International Classification of Diseases and Related Health Problems (ICD). The study protocol was reviewed and approved by the research ethics committee prior to data collection and analysis. This study’s design, analysis, and reporting all adhere to the STROBE (Strengthening the Reporting of Observational Studies in Epidemiology) guidelines [16].

### 2.2. Patient Population

Included patients were those aged 18 years or older, newly diagnosed with stage I to III breast cancer in Alberta, from January 2008 to December 2017, who received at least one cycle of moderately or highly myelosuppressive chemotherapy in the neoadjuvant or adjuvant setting. Patients who were diagnosed with multiple cancers were excluded. Patients were also excluded if they had received G-CSF as part of a clinical study protocol or if they were treated with myelosuppressive.

### 2.3. Study Data

The main outcome of interest was the receipt of either filgrastim or pegfilgrastim during the study period, January 2008 to November 2019. The date of initiation of the first cycle of chemotherapy was considered the study index date. Primary prophylaxis was defined as G-CSF administration within seven days of the start of a chemotherapy cycle. Chemotherapy regimens with high neutropenic risk included AC-T (doxorubicin hydrochloride and cyclophosphamide followed by paclitaxel), DC (docetaxel and cyclophosphamide), ddAC (dose-dense doxorubicin and cyclophosphamide), FEC-D (5-fluorouracil, epirubicin, and cyclophosphamide followed by docetaxel), and TCH (docetaxel, carboplatin, and trastuzumab), whereas those with moderate neutropenic risk were CMF (cyclophosphamide, methotrexate, and 5-fluorouracil), 3-weekly docetaxel, and FEC (5-fluorouracil, epirubicin, and cyclophosphamide) [9].

Demographic information retrieved from the ACR comprised age at treatment initiation and residential postal code. Postal codes were used to derive information on neighborhood-level socioeconomic status, including educational attainment and annual household income, based on 2011 census data, which represented the most recent year of available data. 

### 2.4. Statistical Analysis

Descriptive statistics were used to analyse the baseline demographic- and treatment-related characteristics. To elucidate differences in baseline characteristics between groups, the Wilcoxon rank-sum test was used for continuous variables, whereas Pearson’s Chi-squared test or Fisher’s exact test were used for categorical variables. Additionally, standardized mean difference (SMD) was computed to reinforce comparisons of baseline characteristics, with SMD > 0.1 considered indicative of imbalance. Multivariable logistic regression analyses were performed to assess the likelihood of receiving G-CSF as a binary variable (yes/no) and that of receiving filgrastim over pegfilgrastim. All statistical tests were two-sided, and the significance level was defined a priori as <0.05. All analyses were performed using R.

## 3. Results

### 3.1. Cohort Characteristics

In total, we identified 6662 patients diagnosed with early-stage breast cancer from 2008 to 2017 who received either moderately or highly myelosuppressive (neo)adjuvant chemotherapy (Table 1). Among them, 1492 (22.4%), 3602 (54.1%), and 1568 (23.5%) were, respectively, diagnosed with stage I, II, and III disease. The median age at treatment initiation was 54 (interquartile range (IQR) 46–61) years. The majority (97.7%) of patients received (neo)adjuvant chemotherapy regimens with high neutropenic risk. 

### 3.2. G-CSF Use

Of the entire study cohort, 3801 (57.1%) patients received G-CSF at some point during the chemotherapy treatment trajectory (Table 1). Patients who received G-CSF were more likely to have a younger age, south or Calgary zone of residence, higher neighborhood education, higher neighborhood income, advanced disease stage, chemotherapy with high neutropenic risk, or more recent year of chemotherapy initiation (*p* < 0.001, SMD > 0.1 for each). Among the 3801 patients who received G-CSF, the vast majority received pegfilgrastim only (91.5%; *n* = 3477), whereas the others received either filgrastim only (5.7%; *n* = 217) or both pegfilgrastim and filgrastim (2.8%; *n* = 107). Patients who received filgrastim only were more likely to have a younger age, Calgary zone of residence, higher neighborhood education, advanced disease stage, or more recent year of chemotherapy initiation (*p* ≤ 0.02, SMD > 0.1 for each) (Table 2). Among the patients treated with G-CSF, most did not receive the drug as primary prophylaxis in the first chemotherapy cycle (87.5%; *n* = 3323), but those who did were more likely to receive filgrastim versus pegfilgrastim (*p* < 0.001, SMD = 0.251).

### 3.3. Temporal Trends in G-CSF Use

**Figure 1:**
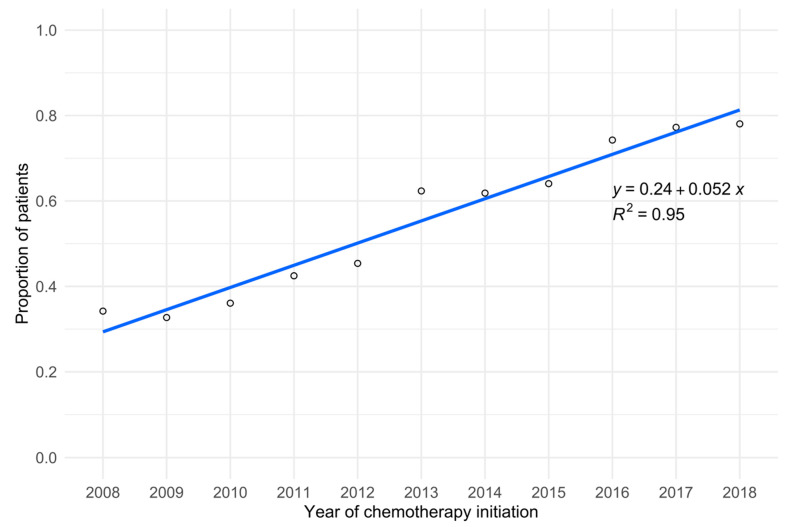
Trends in G-CSF usage by calendar year.

**Figure 2:**
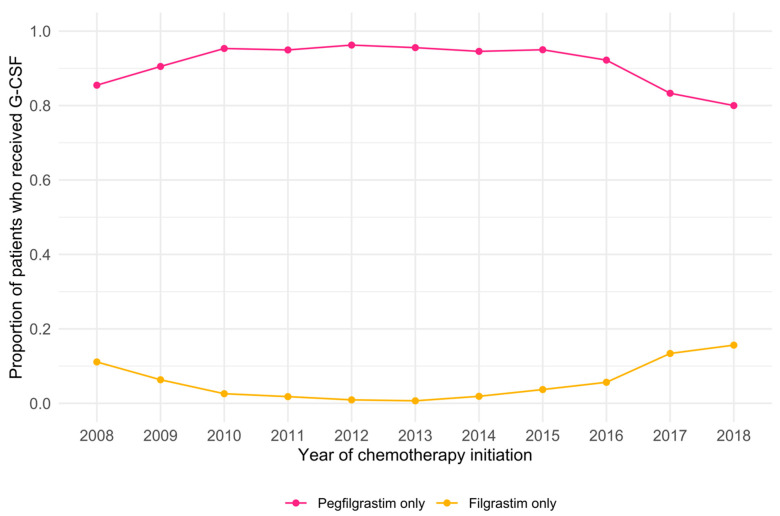
Trends in G-CSF usage by calendar year among patients who received G-CSF, stratified by G-CSF type.

Examining treatment patterns by calendar year, G-CSF use over the 11-year study period appeared to increase linearly from 34.2% in 2008 to 78.0% in 2018 (Figure 1). Among patients who received G-CSF, trends in usage by G-CSF type were more consistent (Figure 2). Even so, there was a marked increase in filgrastim use, accompanied by a proportional decrease in pegfilgrastim use from 2015 to 2018.

### 3.4. Factors Associated with G-CSF Use

In the first multivariable logistic regression model where the outcome was the use of any G-CSF, younger age, south zone of residence, higher neighborhood education, advanced disease stage, receipt of chemotherapy regimens with high neutropenic risk, and more recent chemotherapy initiation were factors that significantly correlated with greater odds of G-CSF use (*p* < 0.01 for all) (Table 3). In the second model where the outcome was the use of filgrastim only versus pegfilgrastim only, younger age, Calgary zone of residence, receipt of chemotherapy regimens with moderate neutropenic risk, more recent chemotherapy initiation, and G-CSF use as primary prophylaxis for the first chemotherapy cycle emerged as statistically significant factors associated with greater odds of filgrastim use (*p* ≤ 0.02 for all) (Table 4).

## 4. Discussion

This was a large retrospective study of a population-based cohort of patients with early-stage breast cancer to describe the use of G-CSF over an 11-year period in Alberta, Canada. Overall, we observed that G-CSF was not used consistently in the setting of myelosuppressive chemotherapy with a high or moderate risk of FN, where only approximately half of the study cohort had a record of at least one G-CSF prescription. In most of these cases, G-CSF was not used as primary prophylaxis for the first chemotherapy cycle. Despite guidelines that recommend primary G-CSF prophylaxis for chemotherapy regimens with an FN risk of >20%, other longitudinal real-world evidence studies have also reported an underutilization of G-CSF in patients with cancer, including patients with non-metastatic breast cancer at high risk of FN based on chemotherapy and patient-related factors [17]. 

A retrospective study by Fine et al. included 395 patients who initiated G-CSF in the oncology outpatient setting between January 2008 and January 2009 in the Canadian provinces of Ontario and Quebec [14]. Overall, 42% of patients received G-CSF as primary prophylaxis. Of the patients who initiated G-CSF, 44% were treated with pegfilgrastim in Ontario compared to only 2% in Quebec, where pegfilgrastim was not covered by provincial health insurance. The reported differences in the rates of G-CSF use as primary prophylaxis between our study (12.5%) and that of Fine et al. (42%) might be attributable to methodological reasons, such as the sample size and observational period, in addition to the provincial coverage of these medications and other factors that would influence the rates of overall use. As demonstrated in the Fine et al. study, the systemic barriers to G-CSF access and delivery may contribute to lower-than-expected usage rates. As funding for supportive care medications in Alberta, including G-CSF, is not currently covered by the cancer care budget, access to these medications is reliant upon self-pay or private insurance. According to a pan-Canadian analysis of prescription drug coverage by the Canadian Alliance for Sustainable Health Care, 30% of Alberta residents below 65 years of age were not enrolled in public or private coverage in 2017 [18]. Furthermore, physicians may have varying levels of knowledge regarding G-CSF guidelines and understanding of the FN risks associated with common chemotherapy regimens [19]. Improved physician awareness about G-CSF use may also lead to higher quality of care.

Even though G-CSF use was suboptimal overall, we observed sizeable and consistent growth in G-CSF use from 2008 to 2018, both graphically and through regression analysis. These temporal trends were concordant with the analysis of a US Medicare population of older women receiving adjuvant chemotherapy for early-stage breast cancer between 2002 and 2012 [20]. Such positive progress on the use of G-CSF prophylaxis for at risk groups could be due to the improved education of patients and providers, regarding the utility of G-CSF in improving treatment outcomes and its positive impact on health-related quality of life, given the high burden of FN and its associated complications. Prior to the availability of biosimilar G-CSF agents, primary prophylaxis with G-CSF may be less cost-effective for FN prevention in breast cancer, when compared to secondary prophylaxis [21]. However, a recent cost-effectiveness analysis of biosimilar filgrastim found primary prophylaxis to be cost-effective and supported expanding the use of G-CSF as an effective method of reducing unnecessary healthcare facility visits, especially in the context of the COVID-19 pandemic [22].

The introduction of biosimilars, such as Grastofil, may further improve G-CSF use. This biosimilar of filgrastim was introduced in Alberta in 2017, toward the end of our study period. While the change in overall G-CSF use was linear throughout our study period, we observed an increase in the proportion of patients receiving filgrastim, compared to pegfilgrastim around 2017. This may indicate a shifting treatment paradigm, attributed to the introduction of Grastofil and to the fact that some private insurance plans implemented a preferential listing of Grastofil over pegfilgrastim in fall 2016. The role of biosimilars in improving access by reducing cost-associated barriers is well-supported. The launch of biosimilar filgrastim in Europe was associated with initial cost savings of 14–27%, increased access, and decreased rates of FN-related hospitalization [23,24,25]. Moreover, numerous studies have suggested that the most cost-efficient approach to reducing the incidence of FN in chemotherapy-treated patients is through use of biosimilars and that savings could be used to expand access or reallocated to other anti-neoplastic therapy options on a budget-neutral basis [26,27]. Significant cost savings and increased access could potentially be realized for health systems that prioritize the use of biosimilars [22]. Nevertheless, the association between biosimilars and trends in G-CSF use in the Canadian context remains unclear and can be a compelling issue for additional investigation, including the extent to which FN-related hospitalizations have been impacted due to increased utilization of G-CSF prophylaxis.

We found certain patient factors to be associated with the increased use of G-CSF and the preference of one G-CSF type over another. Of the demographic characteristics analysed, zone of residence, neighborhood education, and age were significant predictors of G-CSF use. Since southern-most zones of residence are also those with relatively higher population density, we hypothesize that geographic barriers to care may have contributed to the variations in the rates of G-CSF use across the province. Neighborhood education may serve as a surrogate measure of health literacy, which has well-established links with access to care and health outcomes [28,29]. Low health literacy is also a recognized barrier to effective patient care that is especially prevalent in older adults [30]. Some factors associated with G-CSF receipt, namely having advanced disease and receiving chemotherapy regimens with higher FN risk, were concordant with the findings from previous studies [19,31]. Our findings may inform the development of educational tools that promote the appropriate use of G-CSF and policies that enhance access to important supportive therapies in cancer.

Our study design has inherent limitations. We focused on patients with breast cancer due to the high incidence of FN in this population, but we inevitably excluded other patients treated with myelosuppressive chemotherapy, who may benefit from G-CSF. We extracted data for G-CSF and chemotherapy administration from pharmacy dispensing records, rather than usage records; thus, we could not assess G-CSF use after the first cycle of chemotherapy. Lastly, our retrospective approach to data collection may have introduced unknown variables with the potential to confound the relationship between patient factors and G-CSF use.

## 5. Conclusions

There was a consistent rise in G-CSF use in the Alberta healthcare system from 2008 to 2018, yet an appreciable proportion of patients with breast cancer for whom G-CSF prophylaxis is recommended did not receive it. Opportunities exist to further optimize G-CSF use to align with current supportive care clinical guidelines and to reduce the impact of neutropenia and its associated complications. Characteristics of the patient and their cancer treatment should be carefully taken into consideration when planning strategies for supportive care. Potential directions for future research include expanding the evaluation of G-CSF use into other cancer sites and unpacking the impact of biosimilars on improving G-CSF access at a pan-Canadian level.

## Figures and Tables

**Table 1:** Baseline characteristics of the study population, stratified by G-CSF receipt.

Characteristic	Overall (*n* = 6662)	G-CSF Receipt		*p* Value	SMD
		No (*n* = 2861)	Yes (*n* = 3801)		
**Age at chemotherapy initiation, y** (*n* = 6662)	54 (46, 61)	54 (47, 62)	53 (45, 61)	<0.001 **	0.144 ^†^
<45	1394 (20.9%)	518 (37.2%)	876 (62.8%)	<0.001 **	0.137 ^†^
45–54	2139 (32.1%)	921 (43.1%)	1218 (56.9%)		
55–64	2089 (31.4%)	926 (44.3%)	1163 (55.7%)		
≥65	1040 (15.6%)	496 (47.7%)	544 (52.3%)		
**Rurality of residence** (*n* = 6662)				0.21	0.031
Rural	1327 (19.9%)	590 (44.5%)	737 (55.5%)		
Urban	5335 (80.1%)	2271 (42.6%)	3064 (57.4%)		
**Zone of residence** (*n* = 6662)				<0.001 **	0.277 ^†^
Calgary	2717 (40.8%)	1015 (37.4%)	1702 (62.6%)		
Central	722 (10.8%)	290 (40.2%)	432 (59.8%)		
Edmonton	2283 (34.3%)	1156 (50.6%)	1127 (49.4%)		
North	566 (8.5%)	282 (49.8%)	284 (50.2%)		
South	374 (5.6%)	118 (31.6%)	256 (68.4%)		
**Neighborhood education quartile** (*n* = 6613)				<0.001 **	0.114 ^†^
Lowest	1654 (25.0%)	760 (45.9%)	894 (54.1%)		
Second	1653 (25.0%)	751 (45.4%)	902 (54.6%)		
Third	1652 (25.0%)	660 (40.0%)	992 (60.0%)		
Highest	1654 (25.0%)	666 (40.3%)	988 (59.7%)		
**Neighborhood income quartile** (*n* = 6617)				<0.001 **	0.109 ^†^
Lowest	1653 (25.0%)	736 (44.5%)	917 (55.5%)		
Second	1654 (25.0%)	766 (46.3%)	888 (53.7%)		
Third	1655 (25.0%)	682 (41.2%)	973 (58.8%)		
Highest	1655 (25.0%)	655 (39.6%)	1000 (60.4%)		
**Cancer stage** (*n* = 6662)				<0.001 **	0.276 ^†^
I	1492 (22.4%)	823 (55.2%)	669 (44.8%)		
II	3602 (54.1%)	1464 (40.6%)	2138 (59.4%)		
III	1568 (23.5%)	574 (36.6%)	994 (63.4%)		
**Neutropenic risk of chemotherapy regimen** (*n* = 6662)				<0.001 **	0.138 ^†^
High	6510 (97.7%)	2761 (42.4%)	3749 (57.6%)		
Moderate	152 (2.3%)	100 (65.8%)	52 (34.2%)		
**Year of chemotherapy initiation** (*n* = 6662)				<0.001 **	0.689 ^†^
2008	342 (5.1%)	225 (65.8%)	117 (34.2%)		
2009	483 (7.3%)	325 (67.3%)	158 (32.7%)		
2010	535 (8.0%)	342 (63.9%)	193 (36.1%)		
2011	652 (9.8%)	375 (57.5%)	277 (42.5%)		
2012	705 (10.6%)	385 (54.6%)	320 (45.4%)		
2013	470 (7.1%)	177 (37.7%)	293 (62.3%)		
2014	684 (10.3%)	261 (38.2%)	423 (61.8%)		
2015	843 (12.7%)	303 (35.9%)	540 (64.1%)		
2016	882 (13.2%)	227 (25.7%)	655 (74.3%)		
2017	861 (12.9%)	196 (22.8%)	665 (77.2%)		
2018	205 (3.1%)	45 (22.0%)	160 (78.0%)		

SMD standardized mean difference. Values presented as median (interquartile range) or count (column percentage). ** *p* < 0.01. ^†^ SMD > 0.1.

**Table 2:** Baseline characteristics among patients who received either filgrastim or pegfilgrastim, stratified by G-CSF type.

Characteristic	Overall (*n* = 3694)	G-CSF Type		*p* Value	SMD
		Pegfilgrastim Only (*n* = 3477)	Filgrastim Only (*n* = 217)		
**Age at chemotherapy initiation, y** (*n* = 3694)	53 (45, 61)	53 (45, 61)	51 (43, 58)	0.003 **	0.216 ^†^
<45	851 (23.0%)	786 (22.6%)	65 (30.0%)	<0.001 **	0.451 ^†^
45–54	1173 (31.8%)	1110 (31.9%)	63 (29.0%)		
55–64	1138 (30.8%)	1056 (30.4%)	82 (37.8%)		
≥65	532 (14.4%)	525 (15.1%)	7 (3.2%)		
**Rurality of residence** (*n* = 3694)				0.16	0.103 ^†^
Rural	715 (19.4%)	681 (19.6%)	34 (15.7%)		
Urban	2979 (80.6%)	2796 (80.4%)	183 (84.3%)		
**Zone of residence** (*n* = 3694)				<0.001 **	0.410 ^†^
Calgary	1668 (45.2%)	1533 (44.1%)	135 (62.2%)		
Central	416 (11.3%)	404 (11.6%)	12 (5.5%)		
Edmonton	1084 (29.3%)	1041 (29.9%)	43 (19.8%)		
North	275 (7.4%)	257 (7.4%)	18 (8.3%)		
South	251 (6.8%)	242 (7.0%)	9 (4.1%)		
**Neighborhood education quartile** (*n* = 3670)				0.02 *	0.223 ^†^
Lowest	869 (23.7%)	830 (24.0%)	39 (18.0%)		
Second	884 (24.1%)	840 (24.3%)	44 (20.3%)		
Third	964 (26.3%)	890 (25.8%)	74 (34.1%)		
Highest	953 (26.0%)	893 (25.9%)	60 (27.6%)		
**Neighborhood income quartile** (*n* = 3672)				0.16	0.159 ^†^
Lowest	894 (24.3%)	841 (24.3%)	53 (24.4%)		
Second	868 (23.6%)	819 (23.7%)	49 (22.6%)		
Third	940 (25.6%)	895 (25.9%)	45 (20.7%)		
Highest	970 (26.4%)	900 (26.0%)	70 (32.3%)		
**Cancer stage** (*n* = 3694)				0.28	0.108 ^†^
I	653 (17.7%)	606 (17.4%)	47 (21.7%)		
II	2084 (56.4%)	1966 (56.5%)	118 (54.4%)		
III	957 (25.9%)	905 (26.0%)	52 (24.0%)		
**Neutropenic risk of chemotherapy regimen** (*n* = 3694)				0.12	0.104 ^†^
High	3643 (98.6%)	3432 (98.7%)	211 (97.2%)		
Moderate	51 (1.4%)	45 (1.3%)	6 (2.8%)		
**Year of chemotherapy initiation** (*n* = 3694)				<0.001 **	0.947 ^†^
2008	113 (3.1%)	100 (2.9%)	13 (6.0%)		
2009	153 (4.1%)	143 (4.1%)	10 (4.6%)		
2010	189 (5.1%)	184 (5.3%)	5 (2.3%)		
2011	268 (7.3%)	263 (7.6%)	5 (2.3%)		
2012	311 (8.4%)	308 (8.9%)	3 (1.4%)		
2013	282 (7.6%)	280 (8.1%)	2 (0.9%)		
2014	408 (11.0%)	400 (11.5%)	8 (3.7%)		
2015	533 (14.4%)	513 (14.8%)	20 (9.2%)		
2016	641 (17.4%)	604 (17.4%)	37 (17.1%)		
2017	643 (17.4%)	554 (15.9%)	89 (41.0%)		
2018	153 (4.1%)	128 (3.7%)	25 (11.5%)		
**G-CSF as primary prophylaxis for first chemotherapy cycle** (*n* = 3694)				<0.001 **	0.251 ^†^
No	3233 (87.5%)	3062 (88.1%)	171 (78.8%)		
Yes	461 (12.5%)	415 (11.9%)	46 (21.2%)		

SMD standardized mean difference. Values presented as median (interquartile range) or count (column percentage). * *p* < 0.05. ** *p* < 0.01. ^†^ SMD > 0.1.

**Table 3:** Multivariable logistic regression analysis of predictors of G-CSF use.

Variable	OR for Receiving G-CSF (95% CI)	*p* Value
**Age at chemotherapy initiation, y**		<0.001 ^a^**
<45	Reference	
45–54	0.84 (0.72–0.98)	
55–64	0.76 (0.65–0.89)	
≥65	0.57 (0.48–0.68)	
**Rurality of residence**		0.84
Rural	Reference	
Urban	1.01 (0.83–1.23)	
**Zone of residence**		<0.001 **
Calgary	Reference	
Central	0.97 (0.78–1.22)	
Edmonton	0.59 (0.52–0.67)	
North	0.59 (0.46–0.75)	
South	1.12 (0.87–1.46)	
**Neighborhood education quartile**		0.004 ^a^**
Lowest	Reference	
Second	1.07 (0.91–1.26)	
Third	1.28 (1.07–1.53)	
Highest	1.29 (1.06–1.57)	
**Neighborhood income quartile**		0.12 ^a^
Lowest	Reference	
Second	0.88 (0.75–1.03)	
Third	1.06 (0.90–1.26)	
Highest	1.09 (0.91–1.31)	
**Cancer stage**		<0.001 ^a^**
I	Reference	
II	1.93 (1.69–2.21)	
III	2.60 (2.22–3.05)	
**Neutropenic risk of chemotherapy regimen**		0.009 **
High	Reference	
Moderate	0.57 (0.39–0.82)	
**Year of chemotherapy initiation**		<0.001 ^a^**
2008	Reference	
2009	0.90 (0.67–1.22)	
2010	1.15 (0.85–1.55)	
2011	1.48 (1.11–1.97)	
2012	1.68 (1.27–2.22)	
2013	3.01 (2.23–4.09)	
2014	3.24 (2.44–4.32)	
2015	3.71 (2.82–4.91)	
2016	6.37 (4.80–8.49)	
2017	7.61 (5.71–10.19)	
2018	8.55 (5.67–13.10)	

OR odds ratio, CI confidence interval. ^a^ *p* for trend. ** *p* < 0.01.

**Table 4:** Multivariable logistic regression analysis of predictors of filgrastim use among patients who received either filgrastim or pegfilgrastim.

Variable	OR for Receiving Filgrastim Only vs. Pegfilgrastim Only (95% CI)	*p* Value
**Age at treatment initiation, y**		0.001 ^a^**
<45	Reference	
45–54	0.75 (0.52–1.09)	
55–64	0.98 (0.69–1.40)	
≥65	0.15 (0.06–0.32)	
**Rurality of residence**		0.77
Rural	Reference	
Urban	0.93 (0.57–1.56)	
**Zone of residence**		<0.001 **
Calgary	Reference	
Central	0.40 (0.19–0.78)	
Edmonton	0.42 (0.28–0.61)	
North	0.75 (0.40–1.35)	
South	0.46 (0.21–0.91)	
**Neighborhood education quartile**		0.33 ^a^
Lowest	Reference	
Second	1.30 (0.79–2.14)	
Third	1.82 (1.09–3.08)	
Highest	1.33 (0.75–2.35)	
**Neighborhood income quartile**		0.67 ^a^
Lowest	Reference	
Second	0.78 (0.50–1.22)	
Third	0.59 (0.36–0.95)	
Highest	0.91 (0.56–1.47)	
Cancer stage		0.73 ^a^
I	Reference	
II	0.85 (0.59–1.23)	
III	0.95 (0.62–1.48)	
**Neutropenic risk of chemotherapy regimen**		<0.001 **
High	Reference	
Moderate	2.75 (0.95–6.90)	
**Year of chemotherapy initiation**		<0.001 ^a^**
2008	Reference	
2009	0.53 (0.21–1.29)	
2010	0.21 (0.06–0.59)	
2011	0.14 (0.04–0.40)	
2012	0.08 (0.02–0.28)	
2013	0.06 (0.01–0.24)	
2014	0.17 (0.06–0.44)	
2015	0.36 (0.17–0.81)	
2016	0.58 (0.29–1.25)	
2017	1.54 (0.80–3.18)	
2018	1.81 (0.84–4.09)	
**G-CSF as primary prophylaxis for first chemotherapy cycle**		0.02 *
No	Reference	
Yes	1.44 (0.98–2.07)	

OR odds ratio, CI confidence interval. ^a^ *p* for trend. * *p* < 0.05. ** *p* < 0.01.

## Data Availability

Data will not be shared, due to patient confidentiality, according to the ethics approval for this study.
